# Effects of the neurological wake-up test on clinical examination, intracranial pressure, brain metabolism and brain tissue oxygenation in severely brain-injured patients

**DOI:** 10.1186/cc11880

**Published:** 2012-11-27

**Authors:** Raimund Helbok, Pedro Kurtz, Michael J Schmidt, Morgan R Stuart, Luis Fernandez, Sander E Connolly, Kiwon Lee, Erich Schmutzhard, Stephan A Mayer, Jan Claassen, Neeraj Badjatia

**Affiliations:** 1 Division of Neurocritical Care, Department of Neurology/Neurolsurgery, Columbia University Medical Center, Milstein Hospital 8-300 Center, 177 Fort Washington Ave., New York, NY 10032, USA; 2 Clinical Department of Neurology, Neurological Intensive Care Unit, Medical University Innsbruck, Anichstrasse 35, 6020 Innsbruck, Austria; 3 Division of Neurocritical Care, Department of Neurology, University of Maryland School of Medicine, Shock Trauma Neurocritical Care, 110 S. Paca Street, Baltimore, MD 21201, USA

## Abstract

**Introduction:**

Daily interruption of sedation (IS) has been implemented in 30 to 40% of intensive care units worldwide and may improve outcome in medical intensive care patients. Little is known about the benefit of IS in acutely brain-injured patients.

**Methods:**

This prospective observational study was performed in a neuroscience intensive care unit in a tertiary-care academic center. Twenty consecutive severely brain-injured patients with multimodal neuromonitoring were analyzed for levels of brain lactate, pyruvate and glucose, intracranial pressure (ICP), cerebral perfusion pressure (CPP) and brain tissue oxygen tension (P_bt_O_2_) during IS trials.

**Results:**

Of the 82 trial days, 54 IS-trials were performed as interruption of sedation and analgesics were not considered safe on 28 days (34%). An increase in the FOUR Score (Full Outline of UnResponsiveness score) was observed in 50% of IS-trials by a median of three (two to four) points. Detection of a new neurologic deficit occurred in one trial (2%), and in one-third of IS-trials the trial had to be stopped due to an ICP-crisis (> 20 mmHg), agitation or systemic desaturation. In IS-trials that had to be aborted, a significant increase in ICP and decrease in P_bt_O_2 _(*P *< 0.05), including 67% with critical values of P_bt_O_2 _< 20 mmHg, a tendency to brain metabolic distress (*P *< 0.07) was observed.

**Conclusions:**

Interruption of sedation revealed new relevant clinical information in only one trial and a large number of trials could not be performed or had to be stopped due to safety issues. Weighing pros and cons of IS-trials in patients with acute brain injury seems important as related side effects may overcome the clinical benefit.

## Introduction

Titrating sedatives and analgesics to achieve the right balance between deep sedation and wakefulness and to ameliorate patients' comfort in the intensive care unit (ICU) is an integral part of critical care [1]. Over-sedation can lead to prolonged duration of mechanical ventilation and ICU stay and increase the incidence of secondary complications, including nosocomial infections and delirium.

Daily interruption of sedation trials (IS-trials) have been implemented in many surgical and medical ICUs after randomized controlled trials demonstrated that IS decreased the duration of mechanical ventilation, shortened the hospital stay and may, in combination with spontaneous breathing trials, improve outcome in medical intensive care patients [2-6]. Another important issue of IS-trials is that the amount of sedatives and analgesics could also be reduced [2,3]; however, the result of a recent meta-analysis of five randomized controlled trials comparing IS with no interruption in 699 critically ill patients challenges previous findings as a reduction in duration of mechanical ventilation, length of ICU stay or mortality could not be confirmed [7].

IS-trials in patients with acute brain injury are not well studied and the only case series, including 12 patients with traumatic brain injury (TBI) and 9 subarachnoid hemorrhage (SAH) patients, showed that the risk of elevated intracranial pressure (ICP) and low cerebral perfusion pressure (CPP) is evident during these trials [8,9]. Cerebral hypoperfusion and raised ICP may result in an imbalance of energy supply and demand especially for the injured brain and, therefore, aggravate the risk for metabolic distress and brain tissue hypoxia [10-16]. These potential side-effects of IS-trials have not been investigated so far and may limit their clinical benefit in severely brain injured patients. We hypothesized that IS-trials may be harmful for these patients and examined the effects of IS-trials on brain hemodynamic changes, brain tissue oxygen tension and metabolism using multimodal neuromonitoring devices in patients with acute brain injury.

## Materials and methods

### Patients

Between March 2009 and June 2010, 20 consecutive patients with acute brain injury were admitted to the neurological-ICU (NICU) at Columbia University Medical Center and underwent brain multimodality monitoring according to our institutional protocol. At the time monitoring was started, all patients had a Glasgow Coma Scale ≤ 8. In this prospective observational study, we investigated the effect of interruption of sedatives and analgesics on hemodynamic and brain metabolic changes. All interruption in sedation trials were conducted as part of the standard of care in the Neuro-ICU; therefore, consent was not necessary. Data were collected as part of an ongoing prospective database approved by the Institutional Review Board at Columbia University, New York.

### Intracranial monitoring

Multimodality monitoring included at least ICP, cerebral microdialysis, and brain tissue oxygen tension (P_bt_O_2_) measurement and was initiated if (1) it was unlikely that the patient gains consciousness within the following 48 hours, and (2) the patient had a high probability to survive for the next 48 hours. Technical details on probe location, and practical aspects of neuromonitoring have been previously reported and are briefly summarized here [10,11,17]. A CMA-70 microdialysis catheter (CMA/MicrodialysisTM, Stockholm, Sweden), a flexible polarographic Licox Clark-type probe (Licox GMBHTM, Kiel, Germany; Integra-NeurosciencesTM) and a parenchymal ICP monitoring device (Integra-NeurosciencesTM, Plainsborough, NJ, USA) were inserted at the bedside, fixed with a triple-lumen bolt via a frontal approach into the hemisphere deemed at greatest risk for secondary injury (that is, perihematomal or pericontusional tissue, or the ipsilateral anterior watershed zone in lateralized SAH), or in the right frontal lobe in patients with diffuse injury. The location in the white matter was confirmed by brain CT scan immediately after the procedure. Brain metabolic parameters included glucose, pyruvate and lactate concentrations and were analyzed hourly (CMA-600; CMA/MicrodialysisTM).

### Clinical management

Patient care for subarachnoid and intracerebral hemorrhage and TBI conformed to guidelines established by the American Heart Association and the Brain Trauma Foundation [18-20]. In general, hemodynamic and fluid management was targeted to maintain CPP ≥ 60 mmHg, and ICP < 20 mmHg. Adequate sedation and analgesia was performed with a combination of dexmedetomidine, midazolame, propofol and fentanyl. Fever was aggressively treated using intravascular (Celsius-Control-SystemTM, Innercool Therapies, Inc., San Diego, CA, USA) or surface cooling (Arctic-Sun Cooling-SystemTM, Medivance Inc., Louisville, CO, USA) devices. None of the patients were cooled during the IS-trial. Shivering was assessed using the Bedside Shivering Assessment Scale (BSAS) and graded from 0 to 3 points (0 = no shivering noted on palpation of the masseter, neck or chest wall; 1 = shivering localized to the neck and/or thorax only; 2 = shivering involves gross movement of the upper extremities (in addition to neck and thorax) and; 3 = shivering involves gross movements of the trunk and upper and lower extremities) [21]. Treatment of shivering included acetaminophen, buspirone and skin counter warming with forced air at 43°C and magnesium, meperidine and/or dexmedetomidine were used when primary measures failed [21,22].

### Procedure

The goal of the sedation-analgesic regimen in the Neuro-ICU is to utilize the minimal amount of drugs necessary to adequately maintain a safe environment for ventilation, cerebral and systemic hemodynamics while also maximizing the ability to track changes in the neurological examination. Every patient was assessed daily by the treating neurointensivists (JC, SM, KL, NB) for IS-trial eligibility. Interruption was not attempted if one of the following conditions was present: severe hemodynamic instability, ICP > 18 mmHg and the use of sedatives as a primary treatment for ongoing elevated ICP. Other safety concerns included sedative infusions for active seizures, escalating sedative doses due to ongoing agitation or respiratory distress, and evidence of active myocardial ischemia within the last 24 hours. Patients who were eligible underwent interruption of all sedatives and analgesics.

Patients passed the IS-trial if they tolerated sedative interruption for at least 30 minutes (maximum 60 minutes) until awake without exhibiting failure criteria. Patients were monitored by the intensive-care staff or study personnel during the whole trial time. No change was made in fraction of inspired oxygen (FIO2) or positive end-expiratory pressure (PEEP) during the trial. Trained study personnel did neurological assessments, including the Glascow Coma Score (GCS) and FOUR Score, at baseline and after the 60-minute trial or before sedatives and analgesics had to be restarted [23].

Patients failed the IS-trial if they developed sustained anxiety, agitation or pain defined by clinical and cardiopulmonary stress and/or a respiratory rate of more than 35 breaths-per-minute (bpm) for > 5 minutes, an SpO2 < 88% for > 5 minutes, ICP > 20 mmHg and/or CPP < 50 mmHg for > 5 minutes, an acute cardiac dysrhythmia, or ≥ 2 signs of respiratory distress, including tachycardia (> 130 bpm), bradycardia (< 50 bpm), use of accessory muscles, abdominal paradox, diaphoresis or marked dyspnoea. When patients failed an IS-trial, sedatives and analgesics were restarted at half the previous dose and titrated to the desired level of sedation. Microdialysis probes were analyzed, per protocol, before medication was stopped, and in a 60-minute interval thereafter by the ICU staff nurse.

### Data acquisition

The Solar-8000i system utilizing the General Electric Medical Systems Information Technologies' Unity Network^® ^(Milwaukee, WI 53223, USA) was used to capture physiological data. A high resolution data acquisition system (BedmasterEX, Excel Medical Electronics, Jupiter, FL, USA) using the open architecture of the Unity Network^® ^automatically acquired vital sign, alarm and waveform data from all the patient monitoring devices in the NICU. Digital data were acquired every five seconds and recorded in a SQL-database. Waveform data were stored at a resolution of 240 Hz in binary files. The LICOX and brain metabolism data were incorporated into the data acquisition system utilizing the communications (COM) port on the device and is plugged into a serial-to-TCP/IP interface device (Equinox ESP-8, Avocent, Sunrise, FL, USA). Physiological variables including heart rate (HR), arterial blood pressure, respiratory rate (RR), and oxygen saturation (SpO2) were continuously monitored in all patients.

### Statistical analysis

Statistical analysis of pooled data was performed using Mann-Whitney-U, Student-*t-*Test for continuous variables and chi-square test for categorical variables as appropriate. RR, HR, mean arterial pressure (MAP), CPP and P_bt_O_2 _measurements were averaged every 15 minutes and time-locked to the IS-trial. Data are expressed as mean (± SD) or median (IQR) unless otherwise indicated. Time series data were analyzed using a multivariable general linear model (GLM). The model was extended by generalized estimating equations (GEE) using an autoregressor of the first order (AR-1) for model estimation of the data correlation structure of repeated observations within-subjects. Least significant difference (LSD) *post-hoc *comparisons of the estimated marginal means were used to assess differences in outcome variables over time. All statistical analyses were performed using SPSS 19 software (SPSS Inc., Chicago, IL, USA). Differences were considered significant at *P *< 0.05.

## Results

Baseline characteristics are shown in Table 1. Neuromonitoring was initiated at Day 2 after ictus (IQR, 1 to 4) and maintained for 9 days (IQR, 6 to 13). IS trials were performed in all patients, however, not on weekends (*n *= 40), at the day of neuromonitoring probes insertion (*n *= 20), due to technical problems (*n *= 14) and when patients were off sedation (*n *= 19) leaving 82 of 175 days eligible for awakening trials (47%). Interruption of sedation and analgesics was not attempted on 28 days (34%) due to hemodynamic instability (on 7 days), critical ICP values at baseline (on 12 days), and the need for sedatives (on 9 days), resulting in a total of 54 IS-trials being performed.

**Table 1:** Baseline characteristics (*N *= 20)

**Demographics and past medical history**	
Age, y	47 (34 to 56)
Gender (female)	9 (45)
**Diagnosis**	
SAH	14 (70)
ICH	2 (10)
TBI	4 (20)
**Admission neurological examination**	
Admission GCS	**8 (5 to 14)**
Admission Hunt-Hess grade	5 (4 to 5)
1 and 2	2 (10)
3	1(5)
4	3 (15)
5	8 (40)
**Admission physiologic variables**	
APACHE physiologic score	15 (6 to 23)
Systolic blood pressure, mmHg	134 (116 to 160)
**Neuromonitoring**	
Days to Monitoring	2 (1 to 4)
Days with Monitoring	9 (6 to 13)
**Outcome**	
ICU LOS	18 (10 to 26)
Hospital LOS	27 (18 to 37)
Discharge modified Rankin Scale	5 (4 to 5)
< = 3	3 (15)
4	3 (15)
5	9 (45)
Hospital mortality	5 (25)

Values are presented as mean (SD), median (IQR) or number (%). ICH, intracerebral hemorrhage; SAH, subarachnoid hemorrhage; TBI, traumatic brain injury

### Intervention and neurological assessment

**Figure 1:**
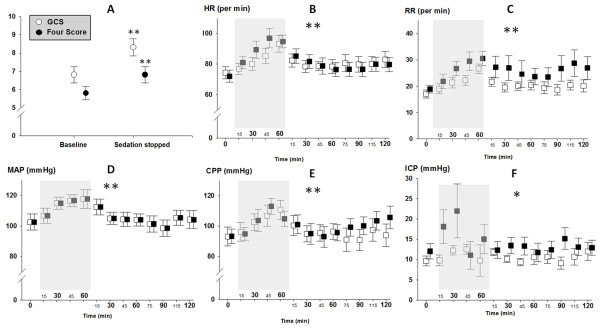
**Clinical and hemodynamic changes during is-trials**. Panel **A **shows median increase of GCS (empty circle) and the Four score (filled circle) during the IS-trial. Panel B-F gives average time course of cardiopulmonary, cerebrovascular parameters and ICP (y-axis) at baseline (time = 0) during the trial (grey square) and over 2 hours following restart of sedation and analgesics (x-axis) in patients where the trial was completed (empty square) and aborted (filled square). *P*-value is given for significant differences of time points (Panel A) or differences of parameters over time (Panel B-F) (** = *P *< 0.001, * = *P *< 0.05). On Panel **B-F **error bars represent means and one standard error of *n *= 54 individual trials. CPP, cerebral perfusion pressure; HR, heart rate; ICP, intracranial pressure; MAP, mean arterial pressure; RR, respiratory rate.

Thirty-six IS-trials (67%) were fully completed and one-third of all trials (*n *= 18, 33%; 12/20 patients) had to be aborted due to ICP crisis (*n *= 9, 50%), agitation (*n *= 4, 22%), systemic desaturation (*n *= 2, 11%) or a combination of all (*n *= 3, 17%). Trial failure was not associated with a specific disease entity or a specific time period after brain injury. Detection of a new neurologic deficit occurred in one trial (2%). In this patient an increase in brain lactate and slight decrease in brain glucose (to 1 mmol/L) was noted hours before the IS-trial was performed. At the trial start, patients received a median of two drugs (IQR, 2 to -3) including a combination of dexmedetomidine (*n *= 45, median = 1 μg/kg/h, 0.7 to 1.0 μg/kg/h), midazolam (*n *= 8, median dose = 1 mg/kg/h, 1 to 7 mg/kg/h), propofol (*n *= 43, median dose = 30 μg/kg/h, 25 to 40 μg/kg/h), and fentanyl (*n *= 30, median = 28 μg/h, 25 to 50 μg/h). The median time off sedation, including all trials, was 35 minutes (30 to 40 minutes). An increase in the FOUR Score (Full Outline of UnResponsiveness score) was observed in 50% of IS-trials by three (two to four) points (*P *< 0.05) (Figure 1A).

### IS-trial and hemodynamic changes

Average values of HR, RR, MAP and CPP at baseline, during the trial and two hours after restart of sedation are shown in Figure 1B-E. All parameters significantly increased from baseline (*P *< 0.001) without discriminating completed trials from interventions where sedation had to be restarted early (*P *> 0.6). Heart rate increased from 73 ± 4 bpm to a maximum of 97 ± 6 bpm, respiratory rate from 18 ± 2 to 30 ± 3/minute, MAP from 103 ± 5 to 117 ± 6 mmHg, and CPP 93 ± 5 to 111 ± 6 mmHg (*P *< 0.001, respectively). ICP was below 15 mmHg in all patients at baseline and remained stable in patients where the trial was completed. In patients where the trial had to be stopped early, ICP increased from a baseline of 12 ± 2 to 22 ± 6 mmHg (*P *< 0.05) (Figure 1F). No further increase in ICP was observed when sedation was restarted.

### IS-trial and changes in brain tissue oxygen tension and cerebral metabolism

**Figure 2:**
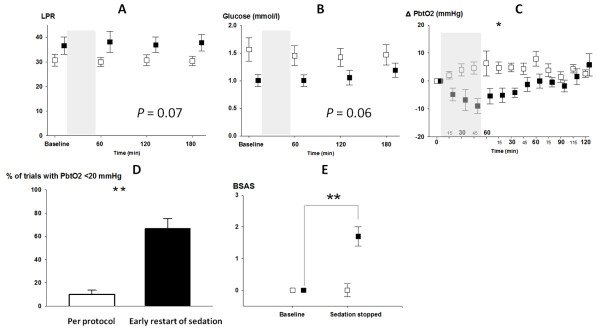
**Changes in brain metabolism, brain tissue oxygen tension and bsas during is-trials**. Average time course of MD parameters before and after the IS-trial (Panel **A-B**). Panel **C **shows average time course of P_bt_O_2 _at baseline (time = 0) during the trial (grey-square) and over two hours following restart of sedation and analgesics (x-axis). Panel **D **gives the percentage of trials with P_bt_O_2 _< 20 mmHg in both groups. In Panel **E **the bedside shivering scale of both groups is shown. Groups are reflected as patients who completed the trial (empty square) and patients who developed a failure criteria (filled square). Error bars represent means and one standard error of *n *= 54 individual trials. *P*-value is given for significant differences (** = *P *< 0.001, * = *P *< 0.05). BSAS, bedside shivering assessment scale; LPR, lactate-pyruvate ratio; P_bt_O_2_, brain tissue oxygen tension.

Brain microdialysate was sufficiently analyzed during 43 IS-trials (78%). Brain tissue lactate, pyruvate, lactate-pyruvate ratio (LPR) and glucose concentration did not change from baseline in both groups (Figure 2A, B). LPR increased by 5% (IQR, 3 to 14%) in 20 trials and decreased by 5% (IQR, 1 to 8%) in 23 trials. We found a tendency to higher overall LPR (*P *= 0.07) and lower overall brain glucose (*P *= 0.06) in patients who failed the trial.

Brain tissue oxygen tension was 28 ± 2 mmHg at baseline and significantly decreased to 19 ± 2 mmHg in patients who developed one of the trial failure criteria (*P *< 0.05) (Figure 2C). In all of these trials a reduction of P_bt_O_2 _compared to baseline was observed, and in 67% brain tissue hypoxia (P_bt_O_2 _< 20 mmHg) occurred (Figure 2D). P_bt_O_2 _did not decrease below 15 mmHg in any patient. In patients who completed the IS-trial, a slight increase in P_bt_O_2 _(*P *= 0.07) was observed. In these trials P_bt_O_2 _was positively correlated with CPP (r = 0.54, *P *< 0.01).

Shivering (BSAS > 0) was observed in half of the trials and more commonly occurred in patients who failed the IS-trial (77% versus 43%, *P *< 0.05) (Figure 2E) [21].

## Discussion

In the present study we evaluated the effect of interruption of sedatives and analgesics on neurologic assessment, hemodynamic changes, brain metabolism and brain tissue oxygen tension in acutely brain injured patients. Our main findings were that IS-trials were not attempted on one-third of eligible days due to safety concerns, and that one-third of performed trials had to be stopped due to a critical increase in ICP and impending brain tissue hypoxia. All IS-trials were associated with cardiopulmonary stress, and detection of a new neurological deficit that led to a change in management occurred in only one trial (2%).

It is important to weigh pros and cons of IS-trials in patients with acute brain injury. Spontaneous awakening trials have been considered safe in medical and surgical ICU patients, but have not been validated for NICU patients [2-6]. In a recent meta-analysis, including 699 critically ill patients, daily sedation interruption was not associated with a reduction in duration of mechanical ventilation, length of ICU stay or mortality, and the authors call for more evidence before IS-trials should be recommended [7]. This may especially hold true for acutely brain injured patients, where an imbalance of energy supply and demand is common and additional stress may result in cerebral metabolic distress and brain tissue hypoxia [10-16]. We observed a sympathetic stress response with increased heart rate, respiratory rate and MAP during all IS-trials, which is in line with a recent report showing an excess of endogenous catecholamines and corticoids during neurologic wake-up tests [24]. Of interest is the observed effect of IS-trials on cerebral oxygen balance. P_bt_O_2 _increased in patients where the IS-trial could be completed with a positive correlation of P_bt_O_2 _and CPP, which may be explained by increased oxygen delivery without excessive energy consumption [25]. In patients who failed the trial, we observed a decrease in P_bt_O_2 _to critical values (< 20 mmHg) in 67% of IS-trials. In parallel, elevation in ICP was observed, which may increase the demand of oxygen and, therefore, decrease brain tissue oxygen tension. Normalization of P_bt_O_2 _after ICP decreased to normal values supports this hypothesis. Increased systemic oxygen utilization may also result in low brain P_bt_O_2 _levels, which is supported by the observation of systemic desaturation in 5 of 18 trials that had to be aborted and the notification of shivering during IS-trials, as noted by the BSAS-score [21]. The BSAS-score increased by two points, reflecting shivering with involvement of the neck, thorax and gross movement of the upper extremities [21], which was common in patients who failed the IS-trial. Shivering can trigger massive increases in systemic resting energy expenditure, and oxygen consumption [21,22], and can potentially increase the risk of brain tissue hypoxia [16]. Therefore, shivering should be effectively treated by pharmacological and non-pharmacological means [26].

A marked increase in ICP and critical decrease in CPP has been previously described in TBI and SAH patients during neurologic wake up trials [8]. Similarly, we found that elevated ICP was the most often reported failure criteria in our trials. Restarting sedation resulted in an ICP decrease to normal values without the need for additional osmotherapy.

Patients who failed the trial had a trend towards higher LPR and lower brain glucose at trial start and during the observation period. This reflects that these patients were already more prone to brain metabolic distress at trial start, which may be a valuable safety consideration of IS-trials in critically ill neurologic patients. Anaerobic metabolism and mitochondrial dysfunction is common after severe head injury and associated with the initial trauma, increased ICP, global cerebral edema after SAH, focal or generalized brain edema, fever, seizures and others [10-16]. Recently, derangement in neuronal signal processing and energy metabolism potentially leading to sustained neuronal depolarization and depression of brain electrical activity have been described after SAH and TBI [27,28]. In these conditions of deranged cerebral metabolism, the human brain may be more vulnerable to systemic and cerebral stress. It is important to mention that ICP increases and P_bt_O_2 _decreases did not result in brain metabolic changes in our patients, most likely due to the short duration of these episodes as all of these parameters were predefined as trial-failure criteria. Without online cerebral hemodynamic and brain oxygenation values IS-trials may result in prolonged episodes of elevated ICP or brain tissue hypoxia which are associated with anaerobic brain metabolism and poor outcome [11,12,29]. For further comparative studies, monitoring ICP and P_bt_O_2 _during IS-trials seems important, whereas microdialysis parameters seem to discriminate at baseline without adding further information during IS-trials.

One of the major rationales in the benefit of daily awakening trials is the additional information gained from a reliable clinical assessment. An increase in GCS and the FOUR score was observed in half of our patients. Evidence of a new focal neurologic deficit was found in only one SAH patient with known cerebral vasospasm who developed motor weakness of the lower limb. Repeated intra-arterial verapamil application resulted in increased perfusion and prevented cerebral infarction.

With limited clinical benefit of IS-trials in NICU patients, a sedation algorithm guided by commonly used sedation scales may be sufficient. However, the problem with this approach is that we still lack validated sedation scales to guide the neurointensivist in the management of acutely brain injured patients. At the end of each IS-trial, sedatives and analgesics were restarted at half the previous dose and titrated to the desired level of sedation. We believe that this strategy is important to prevent over-sedation of our NICU patients, which is a strong predictor for delirium and prolonged ICU stay. We used a fairly advanced sedation regimen, including dexmedetomidine, on our patients which may not be standard in other NICUs, which may have contributed to our findings and also explain differences to positive trials on medical patients [2-6].

Several potential weaknesses of this study deserve mention. The sample size is small and the population heterogeneous; however, a subanalysis, including SAH patients only, did not change our findings. Still, a potential selection bias limits generalizability of our data to all patients with severe brain injury since only poor grade patients that fulfill the inclusion criteria outlined in the methods section underwent multimodal neuromonitoring (that is, GCS < 8). One may argue that hemodynamic deterioration during IS-trials should be expected in this selective patient population; however, these are the patients with highest risk for secondary brain damage (that is, delayed cerebral ischemia (DCI)) where a proper clinical exam may uncover deterioration early. In one-third of our trials, critical ICP elevation was observed even after a specialized clinician considered the patient's condition as safe. Another point why our results should not be generalized is that IS-trials were limited to the neuromonitoring time and we may have missed important trial days. Neuromonitoring was started at Day 2 after ictus and the initial 48 hours may even be more critical for sedation interruption, even resulting in a higher number of side effects and safety concerns than described in this study. By increasing the sample size we would have been able to better describe a hemodynamic and clinical profile of patients where IS-trials are at high risk for being aborted. Another potential bias in this study is the large number of days when IS-trials were not attempted due to weekend days, or where the intervention was not considered safe. We do not know how these patients would have performed; however, based on our results, it is very likely that patients who were excluded from the trial due to hemodynamic or cerebral hemodynamic abnormalities would have deteriorated further during the trial. Another limitation is that we did not measure cerebral blood flow (CBF) and the status of cerebral autoregulation in all patients, however, observed an increase in respiratory rate in all patients. Hyperventilation is associated with cerebral vasoconstriction and decreased CBF and limited energy supply to the brain (oxygen and glucose delivery).

## Conclusions

Interruption of sedation revealed new relevant clinical information in only one trial. A large number of trials could not be performed or had to be stopped due to safety issues. Although serious brain metabolic changes were not observed, related side effects may overcome clinical benefit in severely brain injured patients and the information gained by multimodal neuromonitoring can be used to safely conduct IS-trials in certain patients and disease states. Contrarily, long-term benefits of IS-trials in selected patients with severe brain injury may still prove beneficial. Future studies should evaluate the ability of advanced neuromonitoring techniques to determine patients most suitable for daily sedation interruption.

## Key messages

• Daily interruption of sedation (IS-trials) is considered safe in medical intensive care patients and associated with improved outcome.

• Little is known about the benefit of IS-trials in acutely brain-injured patients.

• IS-trials were associated with cardiopulmonary distress and brain tissue hypoxia and ICP crisis (one-third) in acutely brain injured patients.

• Weighing pros and cons of IS-trials in patients with acute brain injury seems important.

## Abbreviations

AR-1: autoregressor of the first order; BSAS: Bedside Shivering Assessment Scale; bpm: breaths-per-minute; CBF: cerebral blood flow; CPP: cerebral perfusion pressure; DCI: delayed cerebral ischemia; FiO_2_: fraction of inspired oxygen; FOUR score: Full Outline of UnResponsiveness score; GCS: Gasgow Coma Scale; GEE: generalized estimating equations; GLM: general linear model; HR: heart rate; IS: interruption of sedation; ICP: intracranial pressure; ICU: intensive care unit; IQR: interquartile range; LPR: lactate-pyruvate ratio; MAP: mean arterial pressure; NICU: neurological-ICU; P_bt_O_2_: brain tissue oxygen tension; PEEP: positive end-expiratory pressure; RR: respiratory rate; SAH: subarachnoid hemorrhage; SD: standard deviation; SpO_2_: oxygen saturation; TBI: traumatic brain injury.

## Competing interests

The authors declare that they have no competing interests.

## Authors' contributions

RH, NB, SM, JC, ES and SEC conceived of the study, participated in its design and coordination, and helped to draft the manuscript. RH wrote the manuscript. RH, LF, NB, MRS, KL, SM and JC carried out IS-trials. RH, PK and MJS performed the statistical analysis. All authors critically reviewed, drafted and approved the manuscript for publication.

## References

[B1] Hogarth DK Hall J Management of sedation in mechanically ventilated patients Curr Opin Crit Care 2004 16 40 46 10.1097/00075198-200402000-00007 15166848

[B2] Girard TD Kress JP Fuchs BD Thomason JW Schweickert WD Pun BT Taichman DB Dunn JG Pohlman AS Kinniry PA Jackson JC Canonico AE Light RW Shintani AK Thompson JL Gordon SM Hall JB Dittus RS Bernard GR Ely EW Efficacy and safety of a paired sedation and ventilator weaning protocol for mechanically ventilated patients in intensive care (Awakening and Breathing Controlled trial): a randomised controlled trial Lancet 2008 16 126 134 10.1016/S0140-6736(08)60105-1 18191684

[B3] Kress JP Pohlman AS O'Connor MF Hall JB Daily interruption of sedative infusions in critically ill patients undergoing mechanical ventilation N Engl J Med 2000 16 1471 1477 10.1056/NEJM200005183422002 10816184

[B4] Martin J Franck M Sigel S Weiss M Spies C Changes in sedation management in German intensive care units between 2002 and 2006: a national follow-up survey Crit Care 2007 16 R124 10.1186/cc6189 18062820 PMC2246220

[B5] Mehta S Burry L Fischer S Martinez-Motta JC Hallett D Bowman D Wong C Meade MO Stewart TE Cook DJ Canadian survey of the use of sedatives, analgesics, and neuromuscular blocking agents in critically ill patients Crit Care Med 2006 16 374 380 10.1097/01.CCM.0000196830.61965.F1 16424717

[B6] Tanios MA de Wit M Epstein SK Devlin JW Perceived barriers to the use of sedation protocols and daily sedation interruption: a multidisciplinary survey J Crit Care 2009 16 66 73 10.1016/j.jcrc.2008.03.037 19272541

[B7] Augustes R Ho KM Meta-analysis of randomised controlled trials on daily sedation interruption for critically ill adult patients Anaesth Intensive Care 2011 16 401 409 21675059 10.1177/0310057X1103900310

[B8] Skoglund K Enblad P Marklund N Effects of the neurological wake-up test on intracranial pressure and cerebral perfusion pressure in brain-injured patients Neurocrit Care 2009 16 135 142 10.1007/s12028-009-9255-3 19644774

[B9] Helbok R Badjatia N Is daily awakening always safe in severely brain injured patients? Neurocrit Care 2009 16 133 134 10.1007/s12028-009-9262-4 19669605

[B10] Helbok R Ko SB Schmidt JM Kurtz P Fernandez L Choi HA Connolly ES Lee K Badjatia N Mayer SA Claassen J Global cerebral edema and brain metabolism after subarachnoid hemorrhage Stroke 2011 16 1534 1539 10.1161/STROKEAHA.110.604488 21493918

[B11] Helbok R Kurtz P Schmidt JM Stuart RM Fernandez L Malhotra R Presciutti M Ostapkovich ND Connolly ES Lee K Badjatia N Mayer SA Claassen J Effect of mannitol on brain metabolism and tissue oxygenation in severe haemorrhagic stroke J Neurol Neurosurg Psychiatry 2011 16 378 383 10.1136/jnnp.2009.198754 20884670

[B12] Hillered L Vespa PM Hovda DA Translational neurochemical research in acute human brain injury: the current status and potential future for cerebral microdialysis J Neurotrauma 2005 16 3 41 10.1089/neu.2005.22.3 15665601

[B13] Vespa PM Miller C McArthur D Eliseo M Etchepare M Hirt D Glenn TC Martin N Hovda D Nonconvulsive electrographic seizures after traumatic brain injury result in a delayed, prolonged increase in intracranial pressure and metabolic crisis Crit Care Med 2007 16 2830 2836 10.1097/01.CCM.0000295667.66853.BC 18074483 PMC4347945

[B14] Vespa PM Metabolic penumbra in intracerebral hemorrhage Stroke 2009 16 1547 1548 10.1161/STROKEAHA.108.542803 19286575

[B15] Vespa P Bergsneider M Hattori N Wu HM Huang SC Martin NA Glenn TC McArthur DL Hovda DA Metabolic crisis without brain ischemia is common after traumatic brain injury: a combined microdialysis and positron emission tomography study J Cereb Blood Flow Metab 2005 16 763 774 10.1038/sj.jcbfm.9600073 15716852 PMC4347944

[B16] Oddo M Frangos S Maloney-Wilensky E Andrew Kofke W Le Roux PD Levine JM Effect of shivering on brain tissue oxygenation during induced normothermia in patients with severe brain injury Neurocrit Care 2010 16 10 16 10.1007/s12028-009-9280-2 19821062

[B17] Stuart RM Schmidt M Kurtz P Waziri A Helbok R Mayer SA Lee K Badjatia N Hirsch LJ Connolly ES Claassen J Intracranial multimodal monitoring for acute brain injury: a single institution review of current practices Neurocrit Care 2010 16 188 198 10.1007/s12028-010-9330-9 20107926

[B18] Broderick J Connolly S Feldmann E Hanley D Kase C Krieger D Mayberg M Morgenstern L Ogilvy CS Vespa P Zuccarello M Guidelines for the management of spontaneous intracerebral hemorrhage in adults: 2007 update: a guideline from the American Heart Association/American Stroke Association Stroke Council, High Blood Pressure Research Council, and the Quality of Care and Outcomes in Research Interdisciplinary Working Group Circulation 2007 16 e391 413 10.1161/CIRCULATIONAHA.107.183689 17938297

[B19] Bederson JB Connolly ES Jr Batjer HH Dacey RG Dion JE Diringer MN Duldner JE Jr Harbaugh RE Patel AB Rosenwasser RH Guidelines for the management of aneurysmal subarachnoid hemorrhage: a statement for healthcare professionals from a special writing group of the Stroke Council, American Heart Association Stroke 2009 16 994 1025 10.1161/STROKEAHA.108.191395 19164800

[B20] The Brain Trauma Foundation The American Association of Neurological Surgeons The Joint Section on Neurotrauma and Critical Care Critical pathway for the treatment of established intracranial hypertension J Neurotrauma 2000 16 537 538 10937898 10.1089/neu.2000.17.537

[B21] Badjatia N Strongilis E Gordon E Prescutti M Fernandez L Fernandez A Buitrago M Schmidt JM Ostapkovich ND Mayer SA Metabolic impact of shivering during therapeutic temperature modulation: the Bedside Shivering Assessment Scale Stroke 2008 16 3242 3247 10.1161/STROKEAHA.108.523654 18927450

[B22] Badjatia N Strongilis E Prescutti M Fernandez L Fernandez A Buitrago M Schmidt JM Mayer SA Metabolic benefits of surface counter warming during therapeutic temperature modulation Crit Care Med 2009 16 1893 1897 10.1097/CCM.0b013e31819fffd3 19384208

[B23] Wijdicks EF Bamlet WR Maramattom BV Manno EM McClelland RL Validation of a new coma scale: the FOUR score Ann Neurol 2005 16 585 593 10.1002/ana.20611 16178024

[B24] Skoglund K Enblad P Hillered L Marklund N The neurological wake-up test increases stress hormone levels in patients with severe traumatic brain injury Crit Care Med 2012 16 216 222 10.1097/CCM.0b013e31822d7dbd 22179339

[B25] Rosenthal G Hemphill JC Manley G Brain tissue oxygen tension is more indicative of oxygen diffusion than oxygen delivery and metabolism in patients with traumatic brain injury Crit Care Med 2009 16 379 380 10.1097/CCM.0b013e318193265f 19318818

[B26] Choi HA Badjatia N Mayer SA Hypothermia for acute brain injury-mechanisms and practical aspects Nat Rev Neurol 2012 16 214 22 22371279 10.1038/nrneurol.2012.21

[B27] Dreier JP The role of spreading depression, spreading depolarization and spreading ischemia in neurological disease Nat Med 2011 16 439 447 10.1038/nm.2333 21475241

[B28] Hartings JA Bullock MR Okonkwo DO Murray LS Murray GD Fabricius M Maas AI Woitzik J Sakowitz O Mathern B Roozenbeek B Lingsma H Dreier JP Puccio AM Shutter LA Pahl C Strong AJ Spreading depolarisations and outcome after traumatic brain injury: a prospective observational study Lancet Neurol 2011 16 1058 1064 10.1016/S1474-4422(11)70243-5 22056157

[B29] Marmarou A Increased intracranial pressure in head injury and influence of blood volume J Neurotrauma 1992 16 Suppl 1 327 332 1588624

